# Precision TMS through the integration of neuroimaging and machine learning: optimizing stimulation targets for personalized treatment

**DOI:** 10.3389/fnhum.2025.1682852

**Published:** 2025-09-29

**Authors:** Bing Liu, Chunyun Hu, Panxiao Bao

**Affiliations:** ^1^Zhejiang Joint Research Institute of Applied Psychology, Hangzhou, China; ^2^Cixi Seventh People’s Hospital, Ningbo, China

**Keywords:** transcranial magnetic stimulation, neuroimaging, artificial intelligence, precision treatment, personalized medicine

## Abstract

Transcranial Magnetic Stimulation (TMS), a non-invasive neuromodulation technique based on electromagnetic induction, modulates cortical excitability by inducing currents with a magnetic field. TMS has demonstrated significant clinical potential in the treatment of various neuropsychiatric disorders, including depression, anxiety, and Parkinson’s disease. However, conventional TMS targeting methods that rely on anatomical landmarks do not adequately account for individual differences in brain structure and functional networks, leading to considerable variability in treatment responses. In recent years, advances in neuroimaging techniques–such as functional magnetic resonance imaging (fMRI) and diffusion tensor imaging (DTI)–together with the application of machine learning (ML) and artificial intelligence (AI) algorithms in big data analysis, have provided novel approaches for precise TMS targeting and individualized treatment. This review summarizes the latest developments in the integration of multimodal neuroimaging and AI technologies for precision neuromodulation with TMS. It focuses on critical issues such as imaging resolution, AI model generalizability, real-time feedback modulation, as well as data privacy and ethical considerations. Future prospects including closed-loop TMS control systems, cross-modal data fusion, and AI-assisted brain-computer interfaces (BCIs) are also discussed. Overall, AI-driven personalized TMS strategies hold promise for markedly enhancing treatment precision and clinical efficacy, thereby offering new theoretical and practical guidance for individualized treatment in neuropsychiatric and neurodegenerative disorders.

## 1 Introduction

Since its initial description by Barker et al. (1985), Transcranial Magnetic Stimulation (TMS) has attracted widespread attention due to its unique advantage of non-invasively modulating neural activity via electromagnetic induction. With technological advancements, TMS has evolved from single-pulse stimulation to repetitive TMS (rTMS), demonstrating significant clinical efficacy in modulating neuroplasticity and ameliorating brain dysfunction, particularly in the treatment of depression, anxiety, obsessive-compulsive disorder, and post-traumatic stress disorder (PTSD) (Lefaucheur et al., 2014, 2020; O’reardon et al., 2007). Although TMS is associated with high safety and moderate efficacy, its conventional targeting methods–primarily based on the “5-cm rule” or the use of electromyography (EMG) to identify motor evoked potential (MEP) hotspots on the motor cortex, often referred to as “EEG-guided hotspot localization” in a broader sense of electrophysiological guidance–largely overlook inter-individual variations in brain structure and functional connectivity. Consequently, heterogeneity in cortical morphology, functional connections, and white matter pathways among patients limits both treatment response and overall clinical efficacy (Fox et al., 2012; Hanoglu et al., 2023).

To overcome these limitations, neuroimaging modalities such as functional magnetic resonance imaging (fMRI) and diffusion tensor imaging (DTI) have been increasingly employed in TMS research in recent years. Owing to its high spatial resolution and capability for real-time monitoring of brain activity, fMRI not only reveals aberrations in functional brain networks but also provides robust evidence for identifying pathological targets. For instance, several fMRI studies in depression have demonstrated that the functional connectivity between the dorsolateral prefrontal cortex (DLPFC) and the cingulate cortex can predict the therapeutic response to TMS (Hanoglu et al., 2023; Liston et al., 2014). Simultaneously, DTI offers quantitative insights into the integrity and trajectories of white matter fibers, thereby elucidating the structural underpinnings of functional connectivity and aiding in the optimization of TMS stimulation pathways and target selection (Kubicki et al., 2007).

The integration of multimodal neuroimaging data–such as fMRI, DTI, and EEG–enables the construction of individualized brain network maps, making it possible to precisely identify optimal stimulation targets. In the era of big data, machine learning and AI techniques have shown immense potential in processing and analyzing large-scale neuroimaging and clinical datasets. These approaches can predict TMS treatment response, optimize stimulation parameters, and even facilitate real-time feedback modulation (Chekroud et al., 2016; Drysdale et al., 2017; Siddiqi et al., 2020). For instance, algorithms such as support vector machines (SVM), random forests, and deep learning have been successfully applied to identify neuroimaging features associated with TMS response (Orrù et al., 2012). Moreover, recent studies indicate that AI algorithms can automatically adjust TMS coil positioning and, through real-time neuroimaging feedback, optimize the stimulation target to enhance treatment efficacy (Tubbs and Vazquez, 2024).

Despite the promising theoretical and preliminary clinical outcomes of integrating neuroimaging with AI for precision TMS, several challenges hinder its widespread adoption. First, the diversity of imaging platforms and data acquisition protocols may limit the generalizability of AI models (Thut et al., 2011). Second, the high inter-individual variability in brain anatomy and dynamic neural activity increases the complexity of individualized neuromodulation (Brunoni et al., 2017). Lastly, issues surrounding data privacy protection and ethical considerations require further exploration (O’reardon et al., 2007; Rossi et al., 2009). In response to these challenges, emerging research is exploring closed-loop TMS control systems, cross-modal data fusion techniques, and AI-assisted BCIs as potential future directions.

To systematically address these issues, this review introduces an integrative framework that links multimodal neuroimaging, computational modeling, and AI-driven closed-loop control into a coherent precision TMS workflow. Specifically, fMRI is employed to capture individualized abnormal brain network patterns; diffusion tensor imaging (DTI) combined with finite element modeling (FEM) guides the modulation of these aberrant neural circuits through individualized electric field simulations; and during treatment, high-temporal-resolution modalities such as EEG or MEG, coupled with AI algorithms, enable dynamic adjustment of stimulation parameters in real time. This stepwise framework–diagnosis (fMRI) → guidance (DTI/FEM) → closed-loop optimization (EEG/MEG + AI)–is designed to bridge group-level prior knowledge with individual-level variability, thereby enhancing both generalizability and personalization in clinical TMS applications.

The objective of this review is to systematically summarize the advances in integrating neuroimaging and AI technologies for precision TMS. We discuss the underlying technical principles and implementation mechanisms related to target selection, parameter optimization, and real-time feedback, while also analyzing current challenges and future trends. The structure of this review is as follows: an introduction to TMS and its conventional applications, a discussion of the progress in neuroimaging and AI for TMS targeting, an examination of the technical and ethical challenges, and finally, a perspective on future directions aimed at facilitating the clinical translation and individualized treatment design of TMS. This review uniquely emphasizes the deep integration of artificial intelligence and machine learning algorithms with multimodal neuroimaging, particularly focusing on how AI drives personalized target optimization, treatment response prediction, and real-time feedback modulation, thereby offering a forward-looking perspective on AI-driven precision neuromodulation.

## 2 Review of the basic principles and clinical applications of TMS

### 2.1 Physical principles and major technical modalities of TMS

Transcranial Magnetic Stimulation (TMS) is a non-invasive neuromodulation technique based on the principle of electromagnetic induction. By generating a brief, high-intensity magnetic field within the stimulation coil, the magnetic flux penetrates the skull and induces an electric current in the cortical tissue, thereby altering the membrane potential of neurons to either activate or inhibit local neuronal activity (Barker et al., 1985).

With technological advancements, TMS has evolved from its initial form of single-pulse stimulation (sTMS) to repetitive TMS (rTMS). Repetitive TMS, which delivers trains of pulses either continuously or intermittently, not only provides immediate modulation of neural activity but also induces long-term neuroplastic changes, thereby enabling sustained therapeutic effects in clinical applications (O’reardon et al., 2007).

In recent years, theta burst stimulation (TBS) has emerged as an efficient TMS modality that has attracted considerable attention. TBS typically employs a series of high-frequency (approximately 50 Hz) pulses over a short period to mimic the brain’s intrinsic theta rhythm. Its stimulation protocols are generally categorized into intermittent TBS (iTBS), which enhances cortical excitability, and continuous TBS (cTBS), which suppresses cortical activity (Cappon et al., 2022; Huang et al., 2005). This approach has demonstrated distinct advantages in clinical practice by achieving lasting neuromodulatory effects within a considerably shorter stimulation period.

### 2.2 Clinical applications and efficacy evaluation of TMS in neuropsychiatric disorders

In the treatment of psychiatric disorders, rTMS has received approval from the United States Food and Drug Administration (FDA) and has shown significant efficacy, particularly in treatment-resistant depression (TRD) (Ilhan and Arikan, 2024; Philip et al., 2018). Multi-center randomized controlled trials have demonstrated that rTMS targeting the left dorsolateral prefrontal cortex (DLPFC) not only effectively alleviates depressive symptoms but also exhibits a favorable side-effect profile (Croarkin et al., 2021). Moreover, individualized TMS protocols that integrate neuroimaging modalities such as functional magnetic resonance imaging (fMRI) or electroencephalography (EEG) hold promise for further enhancing treatment precision and improving long-term patient outcomes (Pettorruso et al., 2021).

A revolutionary breakthrough in this domain is the Stanford Neuromodulation Therapy (SNT, formerly SAINT). This protocol synergistically combines personalized targeting with optimized stimulation parameters: it uses resting-state fMRI to individually target the DLPFC subregion most anticorrelated with the subgenual anterior cingulate cortex (sgACC), and then applies a high-dose, accelerated intermittent theta-burst stimulation (iTBS) protocol over 5 days. A pivotal double-blind randomized controlled trial demonstrated remission rates of nearly 80% in patients with treatment-resistant depression, establishing a new paradigm for rapid and effective TMS therapy (Cole et al., 2020).

Beyond depression, the application of TMS is expanding in other psychiatric disorders, including anxiety, obsessive-compulsive disorder (OCD), and post-traumatic stress disorder (PTSD). For example, deep TMS (dTMS) has been employed to modulate the functional activity of the anterior cingulate cortex (ACC) and the caudate nucleus, thereby improving clinical symptoms in patients with OCD (Pettorruso et al., 2021). Additionally, the incorporation of neuronavigation systems for precise targeting has enhanced both the accuracy and efficacy of TMS treatments in conditions such as anxiety disorders.

In the realm of neurological disorders, TMS has also garnered significant attention. Research has shown that rTMS applied to the primary motor cortex (M1) in patients with Parkinson’s disease (PD) can improve motor function and alleviate tremor symptoms (Chung et al., 2020; Yap et al., 2020). In stroke rehabilitation, rTMS has been found to promote functional reorganization of the affected hemisphere and facilitate neuroplastic recovery, thereby accelerating the rehabilitation process (Li et al., 2022; Zhu et al., 2024). Preliminary studies further suggest that TMS may have potential clinical applications in alleviating chronic pain, treating addictive behaviors, and ameliorating cognitive deficits (Kim et al., 2023).

### 2.3 Limitations of current TMS targeting methods and future directions

Despite significant progress in various clinical domains, traditional TMS targeting methods remain constrained by several limitations. For example, the “5-cm rule” is essentially based on population-averaged anatomical landmarks to approximate the location of the DLPFC. Its primary limitation lies in neglecting substantial inter-individual cortical variability–such as differences in gyral and sulcal morphology–that can cause the stimulation site to deviate considerably from the intended functional region (Fox et al., 2013).

To address these shortcomings, an increasing number of studies have begun to explore connectivity-based target selection methods. By employing neuroimaging techniques such as fMRI to assess individual brain network connectivity, researchers aim to provide more precise, personalized TMS treatment protocols (Cash et al., 2021; De Matola and Miniussi, 2025; Fox et al., 2012; Hollunder et al., 2022; Padberg et al., 2021). Moreover, the substantial heterogeneity in cortical morphology, functional connectivity, and white matter pathways among patients has underscored the need for individualized targeting approaches, as standardized methods are insufficient for optimal therapeutic outcomes in certain populations (Ekhtiari et al., 2019; Hollunder et al., 2022).

Importantly, cortical variability cannot be fully addressed by optical navigation systems alone, since they do not account for skull thickness, cerebrospinal fluid layers, or cortical folding patterns, all of which significantly shape the induced electric field distribution. In recent years, finite element modeling (FEM), also referred to as electric-field (E-field) modeling, has emerged as a critical tool for overcoming this limitation. By constructing individualized head models from MRI data, FEM enables precise simulation of the E-field generated by a TMS coil at specific positions. This allows target optimization that is tailored to each patient’s unique anatomy, ensuring that the peak E-field intensity accurately engages the desired cortical region rather than adjacent sulcal walls or cerebrospinal fluid (Balderston et al., 2022; Cao et al., 2024; Windhoff et al., 2013).

Maximizing the use of individual-level information, particularly through advanced connectivity-guided targeting (which forms the mainstream of personalized clinical protocols), is crucial for improving efficacy. In this context, AI has the unparalleled potential to facilitate and further enhance this process. Therefore, in response to these ongoing challenges and the need for deeper individualization, emerging machine learning and artificial intelligence (AI) techniques are being integrated into TMS research. By developing deep neural network models that leverage multimodal data–including fMRI, DTI, and EEG–researchers can predict individual responses to TMS and optimize stimulation parameters, thereby enhancing treatment precision and efficacy (Eleni Karakatsani et al., 2024; Tokatly Latzer et al., 2025). However, the combination of TMS and AI-based strategies also faces several hurdles. First, the integration of multimodal data remains challenging, as effective fusion of data from disparate sources (fMRI, DTI, and EEG) is required to fully capture the characteristics of individual brain networks (Tokatly Latzer et al., 2025). Second, the generalizability of current AI models is limited when applied across different datasets and clinical settings, constraining their reliability and applicability (Ilhan and Arikan, 2024). Additionally, the deep integration of TMS and AI has raised concerns regarding data privacy and ethical compliance, which collectively pose significant barriers to widespread clinical translation (Cappon et al., 2022).

Looking ahead, the future development of TMS technology is expected to focus on enhancing treatment precision and optimizing personalized therapeutic protocols, encompassing both gradual adjustments of targets and parameters across treatment sessions and, more ambitiously, closed-loop adjustments within a single session. The incorporation of real-time neuroimaging feedback and the establishment of closed-loop control systems could enable dynamic monitoring and adjustment of TMS parameters, thereby providing specific advantages over pre-determined targets/parameters by allowing immediate adaptation to a patient’s fluctuating brain state and optimizing neuromodulatory effects in real-time, further improving clinical outcomes (Esposito et al., 2020; Hanoglu et al., 2023). Simultaneously, leveraging big data and advanced AI techniques to refine individualized treatment strategies will provide robust theoretical support for connectivity-based target selection (Mutz et al., 2019). Furthermore, conducting large-scale, multi-center randomized controlled trials will be critical for validating the efficacy and safety of personalized TMS protocols across various neuropsychiatric conditions (Kannampallil et al., 2022; Nyffeler and Müri, 2010).

## 3 Applications and advances of neuroimaging techniques in precise TMS targeting

### 3.1 Overview of neuroimaging techniques and their role in TMS therapy

Neuroimaging employs various non-invasive imaging modalities–including computed tomography (CT), magnetic resonance imaging (MRI), functional MRI (fMRI), diffusion tensor imaging (DTI), and positron emission tomography (PET)–to assess the brain’s structure, function, and connectivity. These techniques are increasingly pivotal in clinical diagnosis, disease monitoring, and precision treatment (Kleimaker et al., 2020; Wang et al., 2025). With continuous improvements in imaging resolution and data processing methods, multimodal neuroimaging has provided a robust foundation for detecting aberrations in brain networks, thereby broadening its application in the precise diagnosis and treatment of neuropsychiatric disorders (Lefaucheur et al., 2024; Marzouk et al., 2020). In the context of TMS therapy, neuroimaging not only offers objective criteria for accurately localizing stimulation targets but also enables the evaluation of dynamic changes in brain functional networks before and after treatment, which is essential for optimizing therapeutic protocols (Kotoula et al., 2023; Xia et al., 2024). Furthermore, the integration of diverse imaging modalities holds the promise of constructing a comprehensive profile of an individual’s brain network, a development that is critical for enhancing the precision and personalization of TMS interventions.

### 3.2 Application of fMRI in optimizing TMS targeting

Functional magnetic resonance imaging (fMRI) remains one of the most widely used modalities for assessing brain function by detecting blood oxygen level-dependent (BOLD) signals that reflect localized neural activity (Kleimaker et al., 2020; Xia et al., 2024). By utilizing both resting-state fMRI (rs-fMRI) and task-based fMRI (task-fMRI), researchers are able to construct functional connectivity maps of the brain and identify key networks such as the default mode network (DMN) and the executive control network (ECN) (Lefaucheur et al., 2024; Peterchev, 2024). In various neuropsychiatric conditions, these networks often exhibit abnormal connectivity patterns that can serve as important indicators for optimizing TMS targeting (Jiang et al., 2024; Kumari, 2024; Marzouk et al., 2020). Recent advancements in individualized fMRI analysis have enabled the precise identification of optimal stimulation sites among patients, which significantly enhances the response rate to TMS therapy. For instance, De Filippis et al. (2024) demonstrated through real-time fMRI monitoring that TMS-induced changes in neural excitability could provide clinicians with immediate feedback for adjusting treatment parameters, thereby achieving more precise modulation. This strategy, which is based on functional connectivity data, offers both theoretical and practical support for the development of personalized TMS treatment protocols (Zhong et al., 2025).

### 3.3 FMRI-based targeting in depression and anxiety: clinical case studies

In the treatment of depression, fMRI has proven instrumental in precise target localization. Wang et al. (2025) reported that resting-state fMRI revealed a strong correlation between the functional connectivity patterns of the prefrontal cortex and the clinical response to TMS, thereby providing robust support for selecting the optimal stimulation target based on individual differences. Moreover, Cash et al. (2021) found that optimizing TMS targeting using fMRI data resulted in an improvement of treatment response rates by over 30% compared to conventional targeting methods, further validating the feasibility of connectivity-based approaches. In the realm of anxiety disorders, Vaccarino et al. (2024) observed significant aberrant functional connectivity between the prefrontal cortex and the amygdala during task-based fMRI, suggesting a central role of these regions in the pathophysiology of anxiety (Gottschalk and Domschke, 2017). Guided by these findings, researchers have used fMRI data to direct TMS protocols, with outcomes indicating that enhanced functional connectivity between the left DLPFC and the amygdala is closely associated with improvements in anxiety symptoms, thus providing a novel perspective for personalized treatment (Coppola et al., 2019). Collectively, these clinical cases underscore the substantial value of fMRI not only in identifying pathological network abnormalities but also in determining precise TMS stimulation targets, thereby increasing treatment response and efficacy and laying a solid foundation for the future development of individualized TMS therapies.

### 3.4 DTI and its role in analyzing white matter connectivity

In recent years, diffusion tensor imaging (DTI) has made significant strides in neuroimaging and has become a core tool for studying white matter structure and connectivity. DTI measures the anisotropic diffusion of water molecules, thereby reconstructing the three-dimensional trajectories of white matter fibers and providing crucial insights into structural connectivity between different brain regions (Aydogan et al., 2025). This technique has been pivotal in research on neurodegenerative diseases such as Alzheimer’s disease, psychiatric disorders like depression and schizophrenia, as well as brain injuries (Luigjes et al., 2019). For example, DTI studies in Alzheimer’s disease have revealed significant reductions in the integrity of white matter fibers in the corpus callosum, internal capsule, and frontal regions, which may serve as potential biomarkers for early diagnosis (Amlien and Fjell, 2014; Clark and Werring, 2002). In addition, DTI has demonstrated abnormal patterns of white matter damage in key pathways, such as those within the fronto-limbic network, in both depression and schizophrenia, findings that are likely related to deficits in emotional regulation and cognitive function (Repple et al., 2023; Xu et al., 2024). Recent studies indicate that the reduction in white matter integrity in schizophrenia is particularly pronounced in regions such as the corpus callosum, cingulum, and corticospinal tract, thereby providing a structural basis for the observed cognitive and affective dysfunctions (Ahdab et al., 2010; Zhong et al., 2025). Similarly, in anxiety and obsessive-compulsive disorder (OCD), decreased white matter fiber density in the prefrontal–amygdala pathway has been identified, offering a structural rationale for selecting TMS targets based on abnormal connectivity (Stoby et al., 2022; Zrenner et al., 2020).

### 3.5 Personalized TMS targeting strategies based on DTI data

Accurate target selection is critical for the therapeutic efficacy of TMS. Although diffusion tensor imaging (DTI) provides valuable insights into white matter connectivity, it must be emphasized that the most established and widely applied personalization strategy for TMS is based on functional connectivity (FC) derived from fMRI. Seminal studies by Fox et al. (2012) have demonstrated that identifying dorsolateral prefrontal cortex (DLPFC) subregions functionally anticorrelated with the subgenual anterior cingulate cortex represents the current gold standard for improving antidepressant response to TMS. In this framework, DTI serves as a complementary tool to validate and optimize stimulation pathways, ensuring that modulation signals propagate efficiently along the most relevant structural tracts (Denche-Zamorano et al., 2023). Studies have shown that, in the treatment of depression, targeting TMS based on DTI-derived connectivity yields significantly higher treatment response rates than traditional anatomical methods (Antal et al., 2022; Garnaat et al., 2018). Furthermore, in patients with Parkinson’s disease, DTI-based analyses of cortico-cortical or cortico-thalamic connections have enabled the identification of more appropriate stimulation sites, thereby improving motor function (Wang et al., 2019; Zhang and Burock, 2020). Optimizing TMS stimulation pathways using DTI not only facilitates more efficient transmission of the stimulation signal but also targets specific pathological networks, thereby further enhancing therapeutic outcomes (Luber et al., 2022; Muir et al., 2022). For instance, Liimatta demonstrated that integrating DTI data with TMS target selection in treatment-resistant depression resulted in a marked improvement in therapeutic efficacy, with post-treatment assessments of white matter plasticity providing objective data for parameter optimization (Aydogan et al., 2025; Morriss et al., 2024).

### 3.6 Clinical applications of DTI and future directions

Diffusion tensor imaging, as an advanced neuroimaging modality, exhibits considerable promise in refining TMS targeting and optimizing stimulation pathways. Personalized TMS protocols that incorporate DTI data not only enhance the precision of target selection but also improve treatment outcomes by optimizing the stimulation route. Future research is expected to focus on integrating DTI with other neuroimaging modalities (such as fMRI) and AI algorithms to achieve more refined target localization and treatment protocol design through big data analysis (Aydogan et al., 2025). Additionally, multi-center, large-scale clinical trials will be crucial for validating and promoting TMS strategies based on DTI, providing high-quality evidence for their clinical application (Morriss et al., 2024). With ongoing advances in the integration and evolution of these technologies, DTI-assisted TMS treatment strategies are anticipated to play a more significant role in precision medicine for neuropsychiatric and other neurological disorders.

## 4 Applications of multimodal neuroimaging data fusion in precision TMS treatment

### 4.1 Background and applications of multimodal neuroimaging data integration in TMS

Multimodal neuroimaging data fusion seeks to integrate information obtained from various imaging modalities–such as functional magnetic resonance imaging (fMRI), diffusion tensor imaging (DTI), electroencephalography (EEG), and magnetoencephalography (MEG)–to comprehensively elucidate the interrelationships between brain structure and function (Pszczolkowski et al., 2022). Each modality offers distinct advantages: fMRI captures patterns of neural activity, DTI focuses on delineating the structural connectivity of white matter fibers, while EEG and MEG provide high temporal resolution of neural dynamics (Kuhn et al., 2025). By synthesizing these multimodal datasets, researchers can achieve a more holistic representation of the brain, which is critical for the diagnosis and personalized treatment of neuropsychiatric disorders. In recent years, the application of multimodal imaging in neuromodulation has expanded considerably. Notably, integrating fMRI with DTI data has enabled the precise localization of aberrant functional networks in patients with depression, thereby providing individualized TMS targets that significantly enhance therapeutic outcomes and promote neural plasticity (Mcnabb et al., 2025; Raymond et al., 2022; Yang et al., 2024).

### 4.2 Advantages of multimodal data fusion

**FIGURE 1:**
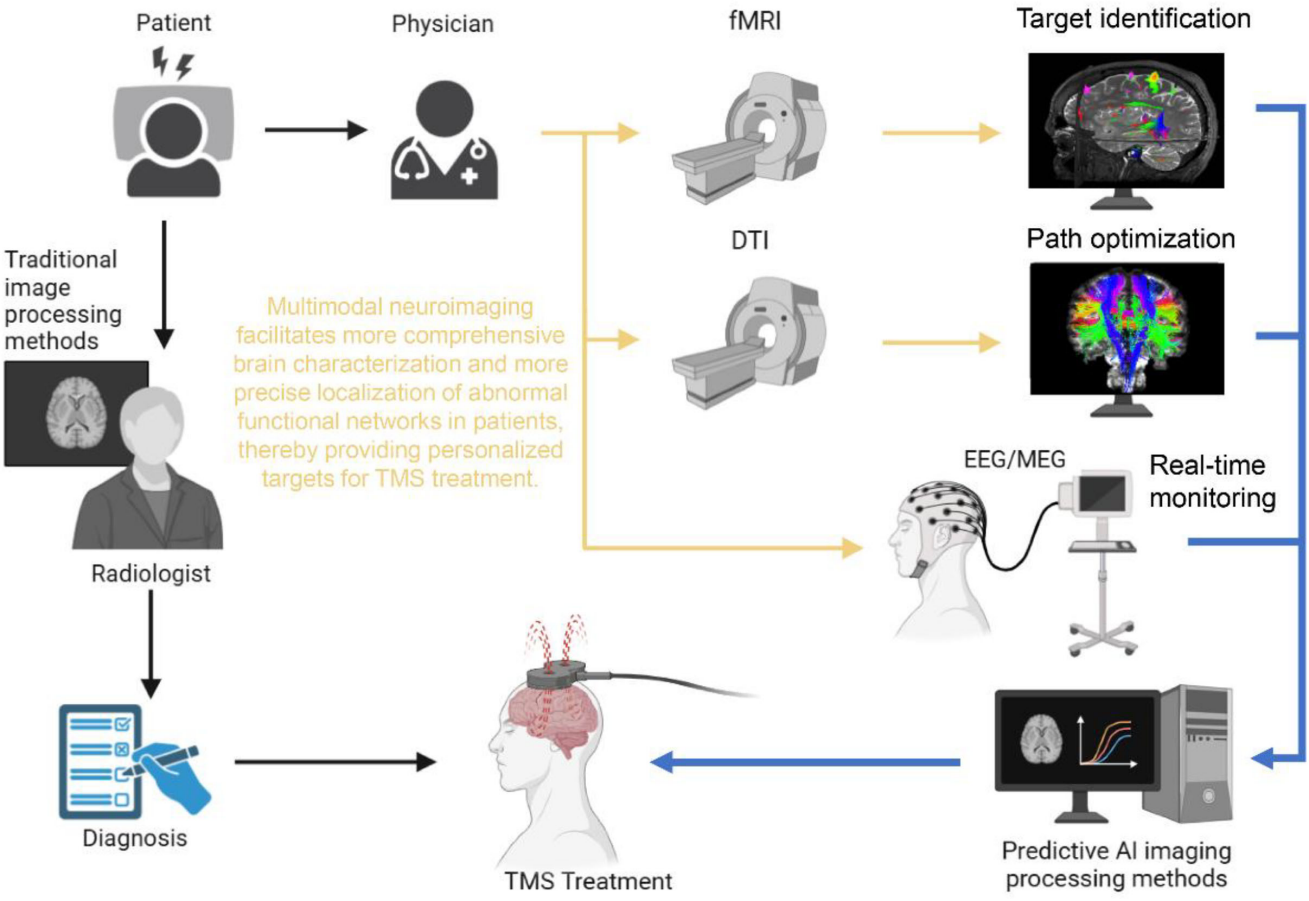
Application of multimodal neuroimaging techniques in TMS target optimization.

The core advantage of multimodal data fusion lies in its ability to leverage the strengths of various imaging techniques to obtain a panoramic understanding of brain structure and function. As depicted in Figure 1, this approach integrates structural connectivity (via DTI), functional activation (via fMRI), and dynamic neural oscillations (via EEG) to map both spatial and temporal dimensions of brain activity, offering a marked improvement over traditional unimodal targeting methods that often fail to capture the dynamic interplay between network nodes. For example, the combination of fMRI, DTI, and magnetic resonance spectroscopy (MRS) has been shown to improve the accuracy of disease diagnosis by precisely identifying white matter lesions in Alzheimer’s disease and predicting disease progression (Gerwig et al., 2012). Moreover, when fMRI is combined with high-temporal resolution data from EEG, the integration of EEG’s millisecond-scale temporal resolution with fMRI’s spatial precision (as shown in the real-time feedback module of Figure 1) enables closed-loop adjustment of TMS parameters based on phase-specific neural states, thereby facilitating the optimization of stimulation parameters and target selection to enhance individualized treatment outcomes (Peterchev, 2024). Additionally, the integration of diverse data sources enriches the input for machine learning and AI algorithms, significantly strengthening the modeling of neural networks. The computational modeling framework (Figure 1) demonstrates how these multimodal inputs are synthesized through machine learning algorithms to generate personalized stimulation protocols, overcoming the limitations of “black-box” open-loop approaches that ignore endogenous brain dynamics. This improved modeling capability enhances the precision of disease progression forecasts and treatment response analyses, ultimately revealing neurobiological differences among patients and providing robust data support for developing more precise TMS stimulation protocols (Cash et al., 2021; Downar et al., 2024).

### 4.3 Challenges in multimodal data fusion

Despite the significant potential demonstrated by multimodal neuroimaging data fusion, its widespread application faces several challenges. First, the heterogeneity of imaging modalities–each with distinct temporal and spatial resolutions as well as signal-to-noise ratios–presents a formidable obstacle to effective data integration (Sui et al., 2023). Second, multimodal data fusion generally relies on complex machine learning algorithms, such as deep learning and graph neural networks, which impose high demands on computational resources and increase data processing and storage costs (Mingyu et al., 2022; Stefanini et al., 2023). Furthermore, the lack of standardized protocols across research institutions for data acquisition and preprocessing leads to variability in data formats and processing pipelines, ultimately compromising the comparability and fusion efficacy of the data (Sui et al., 2023). Lastly, although substantial progress has been made in research settings, the clinical translation of these advanced multimodal imaging techniques for the diagnosis, monitoring, and personalized treatment of neuropsychiatric disorders remains challenging and requires further validation (Liu et al., 2018).

### 4.4 Future prospects and summary

Looking ahead, rapid advancements in artificial intelligence and computational neuroscience are expected to significantly enhance the role of multimodal neuroimaging data fusion in precision medicine. By establishing standardized protocols for data acquisition, optimizing fusion algorithms, and leveraging high-performance computing platforms, future developments will not only overcome current challenges related to data heterogeneity and computational demands but also further improve the accuracy of individualized TMS targeting and treatment efficacy. Large-scale, multi-center clinical trials will be essential for validating and promoting the application of multimodal fusion techniques in neuropsychiatric disorders. Overall, multimodal neuroimaging data fusion offers novel perspectives for elucidating the complex interactions between brain structure and function. Despite the remaining challenges, ongoing technological progress is poised to advance this field to unprecedented levels, ultimately enhancing the precision of TMS treatment in clinical practice.

## 5 Individualized TMS efficacy prediction using artificial intelligence and machine learning

### 5.1 Fundamental principles and applications of machine learning in medical data analysis

In precision TMS treatment, artificial intelligence (AI) and machine learning (ML) play a dual role that bridges population-level generalization with individual-level personalization. At the group level, AI primarily learns from large-scale, multicenter clinical and neuroimaging datasets to identify robust biomarkers predictive of treatment response, thereby constructing models with strong generalizability that overcome the limitations of traditional statistical approaches when handling high-dimensional, heterogeneous data. At the individual level, these generalized models are then applied to each patient’s unique data (e.g., fMRI connectivity patterns, DTI tractography) to generate highly personalized treatment strategies, such as predicting optimal stimulation targets or the likelihood of clinical response. Thus, AI in precision TMS can be summarized as “trained on the group, applied to the individual,” where population-level insights provide the foundation for true individualized optimization.

In recent years, artificial intelligence (AI) and machine learning (ML) techniques have been widely applied in the treatment of neuropsychiatric disorders, with particular promise in predicting individual responses to transcranial magnetic stimulation (TMS) (Chen et al., 2022). Traditional TMS target selection largely relies on anatomical landmarks or group statistical data, which fail to account for individual differences in neural network organization, thereby limiting treatment efficacy (Kuhn et al., 2025). As a data-driven approach, machine learning automatically detects latent patterns within complex datasets and makes predictive inferences. Its workflow typically involves data preprocessing, feature extraction, model training, parameter optimization, and model validation (Dong et al., 2024). In this context, support vector machines (SVMs) are frequently employed for their robust classification capabilities when handling high-dimensional data, distinguishing between responders and non-responders to TMS (Mcnabb et al., 2025). Random forests (RF) enhance model generalizability by aggregating predictions across multiple decision trees (Raymond et al., 2022). Meanwhile, deep learning methods–such as convolutional neural networks (CNNs) and recurrent neural networks (RNNs)–excel at processing neuroimaging and time-series data, capturing intricate neural patterns. Additionally, graph neural networks (GNNs) have demonstrated unique advantages in analyzing connectomic data by modeling relationships among brain regions to predict individual TMS responses (Cash et al., 2021), and ensemble learning techniques improve overall prediction accuracy and stability by integrating multiple model outputs (Zhong et al., 2025). Together, these approaches provide a robust framework for medical data analysis and offer diverse technological options for predicting TMS treatment response.

### 5.2 Multimodal data integration and construction of TMS efficacy prediction models

A key element in predicting TMS efficacy lies in the integration of multiple data sources to comprehensively characterize a patient’s neurobiological status. Researchers typically aggregate multimodal information from neuroimaging (e.g., fMRI, DTI, EEG), clinical assessments (such as depression rating scales, medical history, and medication usage), and genomic data (Kraguljac et al., 2021). After performing feature engineering to extract critical features associated with TMS response–for instance, functional connectivity patterns, cortical thickness, and gene expression levels–machine learning methods (such as SVMs and deep learning) are employed to construct predictive models (Hopman et al., 2021; Nobakhsh et al., 2023). To ensure robust generalization, cross-validation methods (e.g., K-fold cross-validation) are routinely used to assess model performance. Additionally, interpretability techniques such as SHAP (SHapley Additive Explanations) are applied to elucidate key factors in the model’s decision-making process, thereby enhancing clinical interpretability (Dong et al., 2024; Elbau et al., 2023). For example, Arkin et al. (2020) trained a deep learning model using multimodal data to predict the response of patients with schizophrenia to repetitive TMS (rTMS), achieving an area under the ROC curve (AUC) of 0.84, which underscores the advantages of multimodal data in enhancing predictive accuracy (Arkin et al., 2020). Integrating information from diverse sources not only compensates for the limitations inherent in single-modal data but also provides comprehensive support for the development of individualized TMS treatment protocols.

### 5.3 Existing research, model evaluation, and future perspectives

Several studies have already demonstrated significant progress in constructing TMS response prediction models using machine learning techniques. Some investigations have successfully integrated EEG, fMRI, and clinical evaluation data within deep learning frameworks to predict TMS response in patients with schizophrenia, with reported AUC values reaching 0.85–highlighting the critical role of multimodal data in enhancing predictive accuracy (Sachdeva et al., 2023). Furthermore, the application of explainable AI (XAI) techniques in conjunction with fMRI-derived functional connectivity data has not only improved predictive performance but also enhanced the clinical interpretability of the models, enabling clinicians to better understand the underlying decision processes (Herrmann and Ebmeier, 2006). Other studies have found that patients exhibiting the strongest TMS responses often display specific functional connectivity features, and SVM-based classification models built on these neuroimaging markers have further refined the precision of individualized TMS treatments (Jin et al., 2024). Common evaluation metrics for these models include accuracy, AUC-ROC, and the F1-score. Typically, model accuracies range between 80% and 90%, with AUC values exceeding 0.8 considered indicative of excellent predictive performance; higher F1-scores suggest balanced performance across classes, particularly in imbalanced datasets (Mcinnes et al., 2024).

Despite these encouraging advances, challenges remain. The lack of standardized data acquisition across research institutions introduces significant heterogeneity, limiting the generalizability of the models. Furthermore, large-scale, high-quality datasets are still scarce, constraining the potential of deep learning models. Future research should focus on optimizing data integration strategies and exploring privacy-preserving techniques such as federated learning to facilitate cross-institutional data sharing. In addition, combining biomarker data with optimized TMS parameters may pave the way for truly individualized treatment. In summary, as AI and computational neuroscience continue to evolve, multimodal data-integrated TMS efficacy prediction models will provide a more robust technical foundation for the precision treatment of neuropsychiatric disorders and further advance the field of personalized medicine.

## 6 Individualized TMS targeting strategies and clinical prospects based on multimodal neuroimaging and AI integration

### 6.1 Theoretical foundations and technical approaches

In recent years, the integration of neuroimaging techniques–such as functional MRI (fMRI), diffusion tensor imaging (DTI), and electroencephalography (EEG)–with artificial intelligence (AI) has provided novel data-driven methodologies for TMS target localization. Conventional TMS targeting methods predominantly rely on anatomical landmarks or group-level statistical data, which do not sufficiently account for inter-individual differences in brain functional and structural networks, thereby contributing to substantial variability in treatment response (Haxel et al., 2024; Humaidan et al., 2024). To overcome these limitations, current research has explored optimization strategies based on functional connectivity, structural connectivity, and real-time neurodynamics through the application of deep learning, machine learning, and multimodal data fusion. Theoretically, resting-state fMRI can be employed to analyze an individual’s functional connectivity network, enabling the identification of aberrant connections and the subsequent prediction of optimal TMS targets using AI algorithms. Concurrently, DTI provides crucial information regarding the strength of structural connections between different brain regions, which can be leveraged to optimize the TMS stimulation pathway. Moreover, the millisecond temporal resolution of EEG facilitates the capture of dynamic neural responses induced by TMS, allowing for real-time adjustments of stimulation parameters to achieve a personalized treatment approach (Moser et al., 2024; Tubbs and Vazquez, 2024; Vasileiadi et al., 2023). In addition, the application of deep learning models and graph neural networks (GNNs) has enabled the automatic extraction and integration of multimodal neuroimaging data, thereby enhancing both the precision and robustness of individualized TMS protocols.

### 6.2 Integration framework for neuroimaging data and machine learning models

The overall framework for guiding TMS target optimization using multimodal neuroimaging data typically encompasses several sequential stages: data acquisition, preprocessing, feature extraction, model training, and clinical feedback optimization. Initially, multimodal datasets–comprising fMRI, DTI, and EEG–are collected through standardized protocols and preprocessed to ensure compatibility and comparability across modalities. Subsequently, advanced deep learning models (such as CNNs, RNNs, or GNNs) are employed to extract salient features that reflect key indicators of brain functional connectivity, white matter structure, and neurodynamics. These features are then used to construct machine learning models capable of predicting TMS treatment responses in real time or in a pre-treatment setting (Bi et al., 2024; Ran et al., 2022). Based on an individual’s brain network status, these models can recommend optimal stimulation targets and fine-tune stimulation parameters (e.g., frequency, intensity, and pulse patterns) (Liu et al., 2015). This integrated framework not only consolidates methods for analyzing functional and structural connectivity, as well as EEG-guided modulation, but also leverages data fusion and model ensemble techniques to provide a comprehensive depiction of the brain’s multi-level information. For example, Akhonda et al. (2022) demonstrated that analyzing abnormalities in the default mode network and executive control network via machine learning on fMRI data significantly optimized TMS target placement in patients with depression. Similarly, Bi et al. (2024) employed a multitask deep learning framework combined with real-time electric field simulation to automatically recommend optimal TMS coil positioning, thereby further enhancing personalized treatment outcomes. This deep integration of data and modeling not only improves prediction accuracy but also provides actionable clinical decision support.

### 6.3 Success stories and future perspectives for clinical translation

Several studies have already illustrated the significant efficacy of integrating multimodal neuroimaging with AI for TMS target optimization. For instance, a strategy based on fMRI-derived functional connectivity to optimize TMS for depression has successfully improved treatment response rates and has been validated in multi-center trials (Mandal et al., 2023). In another study, the combination of EEG data with AI prediction models enabled real-time TMS modulation, resulting in marked improvements in patients’ emotional states; systems developed on this basis are now progressing toward commercialization and are being applied clinically in the treatment of anxiety and depression (Rahaman et al., 2024). Moreover, DTI-based applications in Parkinson’s disease have shown that by identifying critical white matter fiber tracts, TMS stimulation protocols can be optimized to achieve approximately a 30% improvement in motor function recovery, a method that is gradually being incorporated into individualized TMS treatment guidelines for Parkinson’s disease (Liu et al., 2015).

**FIGURE 2:**
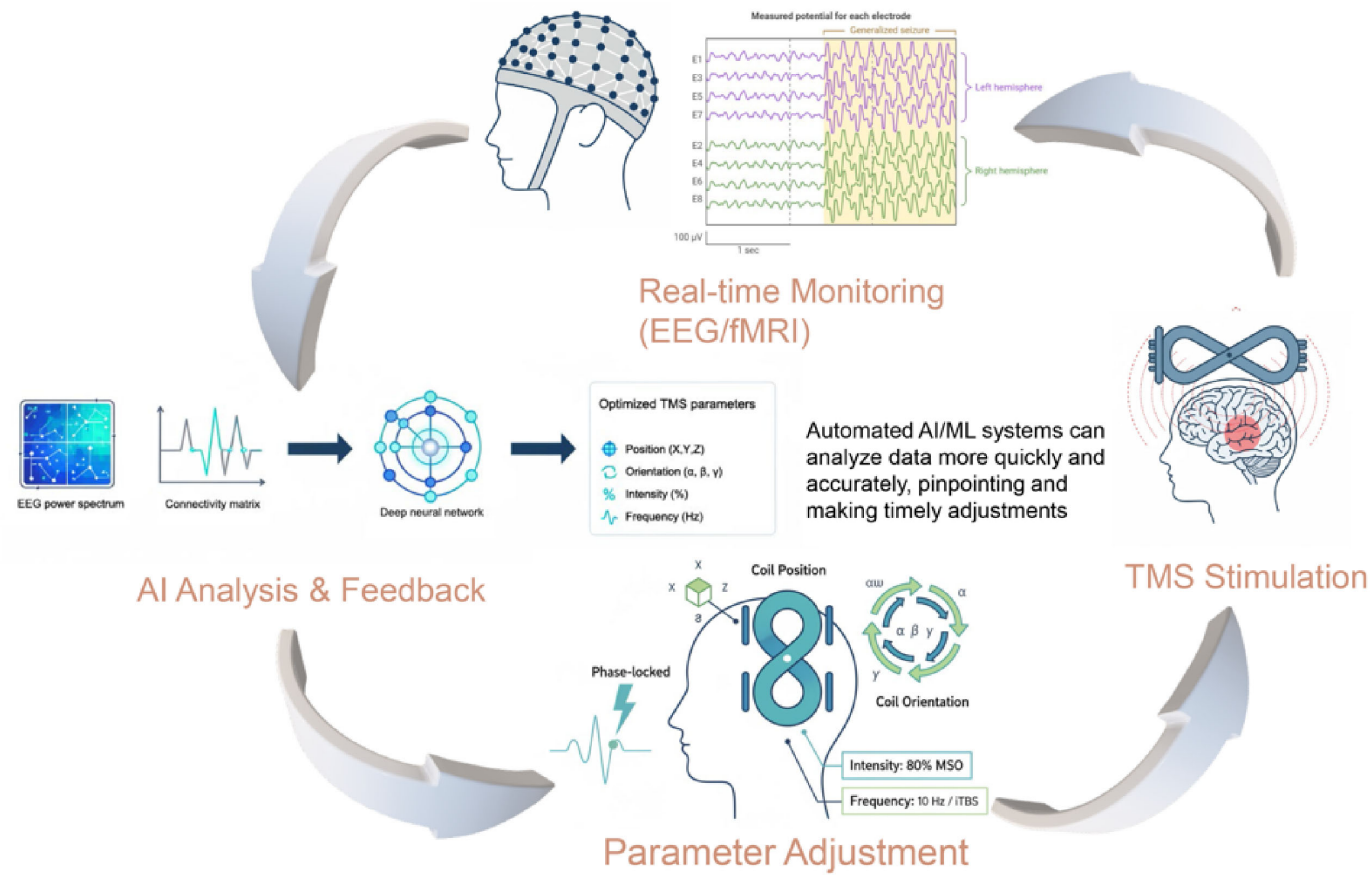
Methodological framework of an AI-driven closed-loop TMS system. The diagram illustrates the operational framework of a closed-loop TMS system for personalized neuromodulation. The process begins with real-time monitoring of neural signals (e.g., EEG, fMRI). These data, represented as inputs like a functional connectivity matrix or EEG power spectrum, are fed into the AI Analysis & Feedback module. The AI model processes this information to generate real-time, optimized recommendations. This output directly guides the Parameter Adjustment module, which demonstrates the dynamic optimization of key TMS parameters. These include the precise coil position (X, Y, Z), orientation (e.g., yaw, pitch, roll), stimulation intensity (% MSO), frequency/pattern (e.g., 10 Hz, iTBS), and timing (e.g., phase-locked to neural oscillations). The adjusted parameters are then applied in the TMS Stimulation stage, completing the adaptive feedback loop.

While these successful examples validate the utility of multimodal data fusion for enhancing TMS efficacy, several challenges remain. Issues related to data heterogeneity, computational resource demands, model interpretability, and cross-institutional data sharing persist. The operational framework for such an advanced system is detailed in Figure 2. This closed-loop architecture demonstrates how real-time neural monitoring (e.g., EEG/fMRI) can feed into an AI model, which in turn provides specific, actionable guidance for dynamically adjusting critical TMS stimulation parameters. As shown in the figure, this includes the coil’s precise position (X, Y, Z) and orientation (yaw, pitch, roll), as well as the stimulation intensity, frequency, and timing relative to neural oscillations. This methodological framework provides a concrete pathway for overcoming current limitations. Future research should aim to further optimize data acquisition and preprocessing standards, develop more efficient and robust fusion algorithms that can process inputs like connectivity matrices and EEG spectra, and employ privacy-preserving techniques such as federated learning to enable large-scale, multi-center data sharing. Moreover, the integration of biomarkers with optimized TMS parameters holds the potential to realize truly individualized and precise treatments, where the closed-loop architecture (Figure 2) could dynamically adapt stimulation protocols based on neural feedback. In summary, as advancements in AI and neuroimaging continue, the clinical translation of multimodal data fusion for TMS target optimization is poised to revolutionize precision medicine for neuropsychiatric disorders.

## 7 Current challenges, limitations, and future prospects: from technical bottlenecks to personalized neuromodulation

### 7.1 Challenges and limitations in current technologies and clinical applications

Despite the substantial promise demonstrated by integrating TMS with neuroimaging and artificial intelligence for optimizing treatment targets and achieving personalized, precise therapy, numerous challenges remain in its clinical application. First, regarding neuroimaging, although fMRI and DTI play pivotal roles in optimizing TMS targets, they still face limitations in spatial and temporal resolution. fMRI is constrained by the indirect nature of the BOLD signal, and DTI may be insufficient in resolving fine white matter fiber connections. Simultaneously, while EEG offers millisecond-level temporal resolution, its relatively low signal-to-noise ratio and significant inter-individual variability can adversely affect the stability and accuracy of TMS target selection (Gebodh et al., 2023; Gu et al., 2023; Seriramulu et al., 2024; Winchester et al., 2023). Furthermore, AI-based TMS prediction models are typically developed using relatively limited clinical datasets, and they lack robust generalizability across multiple centers and diverse patient populations. In addition, deep learning models are often considered “black boxes” due to their opaque decision-making processes, which not only impairs their performance across different pathological states but also diminishes clinicians’ trust and acceptance (Moser et al., 2024; Sun et al., 2023). Data privacy and ethical issues further complicate matters; the high sensitivity of neuroimaging data necessitates strict management protocols during data collection, storage, and sharing to prevent breaches of patient privacy and data misuse (Cappon et al., 2022; Trajkovic et al., 2024). Together, these technological bottlenecks and ethical concerns constitute major barriers to the widespread clinical adoption of personalized TMS treatment.

### 7.2 The need for multi-center validation and standardization of ethical protocols

At the clinical level, the precision treatment strategies that combine TMS with AI require validation through large-scale, multi-center studies. Currently, variations in imaging acquisition protocols, spatial resolution, and preprocessing methods across institutions result in poor comparability of data, thereby limiting the generalizability and reproducibility of AI models in multi-center settings (Haxel et al., 2024; Humaidan et al., 2024; Tubbs and Vazquez, 2024). Moreover, the lack of mature, standardized protocols for multimodal data integration further restricts the stability and scalability of these predictive models. Ethical challenges also remain critical, particularly regarding the extent of patient informed consent and the interpretability of AI-generated treatment recommendations in the formulation of personalized TMS protocols. In vulnerable populations (e.g., patients with autism or schizophrenia), diminished cognitive capacity may further complicate patients’ ability to fully understand and accept AI-derived treatment plans, adding another layer of complexity to ethical decision-making (Dannhauer et al., 2022; Figee et al., 2022).

### 7.3 Future directions and prospects for technological breakthroughs

Looking forward, the future of personalized TMS treatment in neuromodulation is promising, yet it demands breakthroughs in technology, data integration, and ethical standards. The development of closed-loop TMS systems is widely regarded as a critical future direction; by integrating real-time EEG or fMRI monitoring of brain states with AI-driven dynamic adjustment of stimulation parameters, such systems could achieve adaptive control and significantly enhance treatment precision and flexibility (Duprat et al., 2025; Oliver et al., 2024). Additionally, the advancement of real-time feedback control systems, which leverage fMRI data and deep learning algorithms to automatically analyze brain signal changes during treatment and adjust stimulation patterns accordingly, may further refine individualized therapy by closely matching the patient’s dynamic brain network state (Sitaram et al., 2012; Tervo-Clemmens et al., 2023). Future research is also anticipated to focus on the deep fusion of multimodal data–including fMRI, DTI, EEG, PET, and genetic information–using advanced algorithms such as graph neural networks (GNN) or Transformer models to construct accurate, individualized brain network models that can robustly inform TMS target selection (Cappon et al., 2022; Fujimoto et al., 2024). Clinically, as standardization of data acquisition, cross-center collaboration, and large-scale validation advance, it is expected that within the next 5–10 years, AI-driven personalized TMS treatment strategies will be incorporated into standard clinical guidelines and achieve breakthrough outcomes in treating psychiatric disorders, neurodegenerative diseases, and pain management (Lin et al., 2020).

### 7.4 Long-term prospects for personalized and precise neuromodulation

As AI and neuroimaging technologies continue to mature, personalized TMS treatment is poised to usher in a transformative era in neuromodulation. Precision psychiatry models built on neuroimaging and genetic data will enable the prediction of optimal TMS parameters for individual patients, thereby facilitating truly personalized medical interventions. Moreover, the development of AI-assisted brain-computer interfaces (BCIs) is expected to offer new avenues for neurorehabilitation, by potentially synergizing with TMS interventions. For instance, BCIs could be used to detect a patient’s volitional motor intent (e.g., in stroke rehabilitation), which then triggers or modulates TMS stimulation to reinforce the desired neural pathway activation, thereby enhancing motor recovery in stroke patients through targeted plasticity induction. Overall, although current challenges persist in terms of technology, ethics, and clinical validation, ongoing standardization of data collection, multi-center large-scale trials, and the emergence of advanced algorithms are anticipated to drive the full clinical translation of personalized, precision neuromodulation. This evolution will likely propel the treatment of neuropsychiatric disorders into a new era (Moon et al., 2020).

## 8 Discussion

This review systematically examined the progress in applying neuroimaging techniques and artificial intelligence for precision TMS treatment, with a particular focus on the role of multimodal neuroimaging (e.g., fMRI, DTI, and EEG) in elucidating individual variations in brain structure and function, as well as on the application of machine learning and deep learning methods for TMS target localization, treatment response prediction, and real-time feedback modulation. The evidence indicates that the integration of these technologies provides robust data support and a solid theoretical foundation for individualized, precise TMS treatment, thereby significantly overcoming the limitations inherent in conventional anatomical targeting methods (Cao et al., 2021; Zrenner and Ziemann, 2024).

Multimodal neuroimaging can reveal abnormal brain network patterns at different levels. fMRI captures functional connectivity through the BOLD signal, DTI provides insights into the integrity and structural connections of white matter fibers, and EEG offers millisecond-level temporal resolution to track dynamic neural activity. The complementary nature of these imaging modalities enables a more comprehensive modeling of an individual’s brain network (Klooster et al., 2022). For example, a multi-center study by Kim et al. (2023) and Klooster et al. (2022) demonstrated that constructing individual brain network maps using fMRI and DTI data could successfully predict TMS treatment responses in depression, highlighting the substantial potential of multimodal data integration for personalized therapy.

Moreover, artificial intelligence and machine learning techniques have shown significant promise in processing multimodal data and building predictive models. Deep learning, graph neural networks, and ensemble learning methods have been employed to extract critical features from complex neuroimaging and clinical datasets, enabling the prediction of TMS treatment response and optimization of stimulation parameters (Marcu et al., 2020). Kale et al. (2024) reported that a deep learning model integrating EEG, fMRI, and clinical data achieved an area under the ROC curve (AUC) of 0.87, underscoring the advantage of these algorithms in enhancing prediction accuracy. Nevertheless, current models face challenges related to limited sample sizes, the lack of standardized data acquisition protocols across centers, and limited generalizability, which restrict the robustness and clinical interpretability of these predictive systems (Cao et al., 2021).

In addition, data privacy and ethical considerations pose critical challenges for the integration of TMS and AI technologies. High-sensitivity neuroimaging and genetic data require stringent management during collection, storage, and sharing to protect patient privacy and prevent misuse (Kale et al., 2024). Kale et al. (2024) emphasized that ensuring data security and obtaining informed consent are prerequisites for facilitating large-scale, multi-center collaborations that can drive the clinical translation of personalized TMS treatments.

Within the context of interdisciplinary collaboration and technological convergence, the development of closed-loop TMS systems and real-time feedback modulation represents a promising avenue for future treatment paradigms. By continuously monitoring brain states and dynamically adjusting stimulation parameters, such systems could achieve highly adaptive, individualized treatment (Zrenner and Ziemann, 2024). Additionally, the deep fusion of multimodal data and the application of novel machine learning algorithms–such as Transformer models and advanced graph neural networks–are expected to further enhance model prediction accuracy and clinical applicability (Kale et al., 2024). Future research should prioritize improvements in imaging resolution and data quality, the standardization of cross-center data protocols, the enhancement of AI model generalizability and interpretability, and the reinforcement of data privacy safeguards (Cao et al., 2021; Kale et al., 2024).

Overall, the application of neuroimaging and AI in precision TMS has opened new avenues for improving individualized treatment protocols. While these advanced techniques offer promising solutions to overcome the limitations of conventional anatomical targeting, challenges remain in data integration, model generalization, and ethical regulation. Only through interdisciplinary collaboration, continuous technological innovation, and rigorous clinical validation can these cutting-edge methods be fully translated into clinical practice to improve patient outcomes and quality of life. As large-scale, multi-center data sharing and advanced algorithms evolve, personalized TMS strategies based on multimodal data integration are poised to achieve broader clinical application in the treatment of psychiatric disorders, neurodegenerative diseases, and neurorehabilitation.

## 9 Conclusion

This review systematically examines the advancements in transcranial magnetic stimulation (TMS) for the treatment of neuropsychiatric disorders, emphasizing the pivotal roles of neuroimaging technologies–including functional magnetic resonance imaging (fMRI), diffusion tensor imaging (DTI), and electroencephalography (EEG)–alongside artificial intelligence (AI) and machine learning methodologies in precise TMS target localization and personalized therapy. The integration of multimodal neuroimaging data elucidates the intricate networks of individual brain function and structure, while machine learning algorithms enhance the precision and predictive accuracy of TMS treatment protocols. This synergy not only refines target selection and stimulation pathways but also underpins the development of real-time feedback mechanisms and closed-loop TMS systems, thereby facilitating personalized precision therapy.

Furthermore, this article underscores the significance of interdisciplinary research. The convergence of neuroscience, computer science, engineering, and clinical medicine offers novel perspectives to address current challenges, such as data heterogeneity, model generalizability, and ethical considerations regarding privacy. Through standardized data acquisition, inter-center collaboration, and the continuous optimization of advanced algorithms, there is potential to enhance the clinical efficacy of TMS across domains including psychiatric disorders, neurodegenerative diseases, and pain management.

Looking ahead, personalized precision TMS therapy is poised to advance the field of precision psychiatry. With the support of emerging technologies like AI-assisted brain-computer interfaces, its applications may extend to broader areas of neurorehabilitation and functional restoration. In summary, as neuroimaging and AI technologies continue to evolve and multimodal data integration methods mature, individualized neuromodulation strategies based on precise TMS are expected to play an increasingly vital role in clinical translation and widespread application, offering transformative breakthroughs in patient outcomes and quality of life enhancement.
